# CO_2_ in Lyotropic Liquid Crystals: Monoethanolamine-Facilitated Uptake and Swelling

**DOI:** 10.3390/polym10080883

**Published:** 2018-08-07

**Authors:** Sandra Rodríguez-Fabià, Jens Norrman, Johan Sjöblom, Kristofer Paso

**Affiliations:** Ugelstad Laboratory, Department of Chemical Engineering, Norwegian University of Science and Technology (NTNU), 7491 Trondheim, Norway; jens.norrman@ntnu.no (J.N.); johan.sjoblom@ntnu.no (J.S.); kristofer.g.paso@ntnu.no (K.P.)

**Keywords:** lyotropic liquid crystals, CO_2_ capture, phase behavior, PEO-PPO-PEO, MEA

## Abstract

Ternary systems consisting of amphiphilic block copolymers/water/monoethanolamine (MEA) have been studied as potential solvents for carbon capture and storage (CCS). The phase behavior of two poly(ethylene oxide)-poly(propylene oxide)-poly(ethylene oxide) copolymers with average compositions (EO)_8_(PO)_47_(EO)_8_ (L92) and (EO)_3_(PO)_50_(EO)_3_ (L81) have been investigated by cross-polarized visual observation and small angle X-ray scattering (SAXS). The respective ternary phase diagrams have been studied for systems containing MEA and the equivalent systems containing CO_2_-loaded MEA. The presence of MEA loaded with CO_2_ hinders self-association, preventing the formation of liquid crystalline phases. One-phase liquid crystalline regions were found at low MEA concentrations (below 20 wt %) in L92. In the case of L81, only one one-phase region consisting of coexisting lamellar and disordered aggregates was found at 5 wt % MEA. The swelling of the liquid crystalline phases with MEA was investigated along designated dilution lines. The lattice parameters of L92 liquid crystals decrease upon addition of MEA, whereas L81 aggregates show the opposite behavior.

## 1. Introduction

Environmental issues due to global warming have become a public concern in the past several years. One of the main causes of global warming is believed to be CO_2_ emissions, which reached 35.8 billion tones in 2016 [1]. In order to help prevent the effects of global warming, carbon capture and storage (CCS) has been suggested as one of the potential solutions to reduce CO_2_ emissions. The principle behind CCS consists of capturing CO_2_ produced in power plants, transporting it, and then storing it underground for an extended period. The storage locations can be depleted oil and gas reservoirs, deep saline formations, or use of CO_2_ for enhanced oil and gas recovery [2]. Nowadays, the most extensively studied solvents for post-combustion CO_2_ capture are amine solvents, particularly monoethanolamine (MEA) [3,4]. Amine technology was first patented in the 1930s [5], but it is not widely used due to the extensive costs for regenerating the solvent [5,6]. New technologies, such as membranes, ionic liquids, and metal-organic frameworks have been investigated recently as potential alternatives to amine solvents for post-combustion capture [3,4]. In addition, liquid crystals (LCs) have recently been suggested as potential new solvents for CCS [7,8,9,10,11].

**Figure 1:**
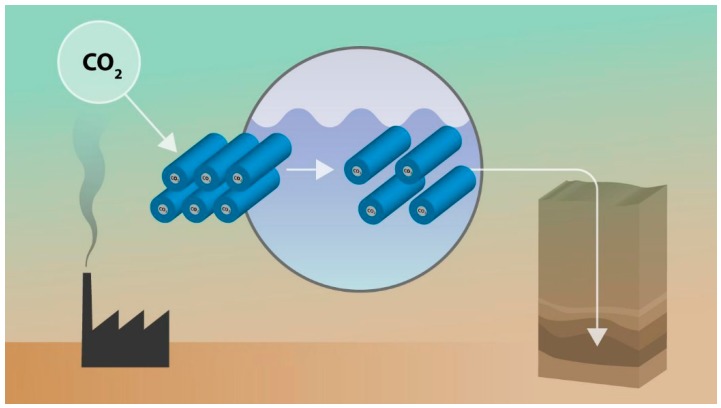
CO_2_ capture, transport, and storage using liquid crystal technology. Illustration by Eivind Vetlesen.

The use of thermotropic LCs for gas sorption was already investigated during the 1990s. In the past few years, there have been several publications related to the use of LCs [12,13,14] and polymeric liquid crystals (PLCs) [15] for gas sorption, although at that time they were not considered as solvents for CO_2_ capture. In 2008, Gross and Jansens suggested a new process using liquid crystals as an alternative technology to capture CO_2_ [7]. Recently, de Groen et al. have investigated the phase behavior of a series of liquid crystals with CO_2_ to assess if the studied liquid crystals are suitable for CO_2_ capture [8,9,10,11,16]. The principle behind the use of thermotropic LCs for CO_2_ sorption is based on the phase transition from the liquid crystalline state to the isotropic liquid state or to the crystalline state. The technology takes advantage of the fast switch between the isotropic and liquid crystalline state triggered by temperature, as well as the difference in solubility of CO_2_ between these two states [7,11,13,14]. In this work, we would like to investigate the potential use of lyotropic liquid crystals for CO_2_ capture and storage. A schematic representation of the CCS process with liquid crystals is illustrated in Figure 1.

Poly(ethylene oxide)-poly(propylene oxide)-poly(ethylene oxide) (PEO-PPO-PEO) are amphiphilic block copolymers also available under commercial names such as Pluronic, or Poloxamer. The hydrophilic/hydrophobic properties of these copolymers can be easily modified by changing the molecular weight of the polymers or the ratio between hydrophilic and hydrophobic blocks. Therefore, PEO-PPO-PEO copolymers are used in many fields such as biomedical applications, detergency, dispersion stabilization, lubrication, and emulsification [17,18,19,20,21].

The phase behavior of PEO-PPO-PEO has been widely studied [22,23,24,25]. When PEO-PPO-PEO copolymers are mixed with water at high polymer concentrations they can form lyotropic liquid crystals, such as lamellar, hexagonal, and cubic phases [22,24]. Svensson et al. experimentally determined the binary phase diagrams of three different Pluronic-water mixtures (Pluronic L62, L92, and L122) [22]. The three polymers presented liquid crystalline phases. Pluronic L92 forms hexagonal, lamellar, and reverse hexagonal phases [22]. In ternary systems, where the copolymers are mixed with two selective solvents, the phase behavior is more complex [25,26]. The effect of cosolvents on the self-assembly of PEO-PPO-PEO copolymers has been investigated by Ivanova et al. [27,28]. In their work, they reported how the phase behavior of these copolymers can be tuned by choosing the appropriate solvent and changing the curvature of the liquid crystalline aggregates.

The principal goal of this work is to find liquid crystalline phases consisting of polymer/water/MEA, and to obtain a better understanding on the effects of the addition of MEA to polymer/water mixtures. Moreover, the changes in phase behavior when CO_2_-loaded MEA is used are also investigated. In this article, we report the ternary phase diagrams of two different Pluronic copolymers (L92 and L81), water, and monoethanolamine (MEA). Similarly, we also report the phase diagrams of Pluronic/water/(MEA + CO_2_). We first present the phase diagrams of Pluronic/water/MEA. Then we show how the phase behavior of the systems changes when MEA loaded with CO_2_ is used. Small-angle X-ray scattering (SAXS) was used to determine the structure of the samples. Finally, we determined the lattice parameters of the liquid crystalline structure and we investigate the swelling of the aggregates when MEA is added to the polymer/water system. The scope of this work was to focus on the liquid crystalline phases, therefore the other regions of the phase diagrams have not been fully explored. The authors acknowledge that to justify any practical application of the studied systems in CO_2_ capture and storage, future work should be carried out to study the phase behavior, CO_2_ diffusion, and phase transition kinetics of the polymer/water/MEA system upon absorption of CO_2_, not just simply doping previously CO_2_-loaded MEA into the polymer/water mixture.

## 2. Materials and Methods

### 2.1. Materials

Pluronic L92 (poly(ethylene oxide)-block-poly(propylene oxide)-block-poly(ethylene oxide)) (PE 9200, *M*_n_ ≈ 3650 g/mol, 20 wt % PEO) was provided by BASF Corporation (Ludwigshafen, Germany). Pluronic L81 (PE 8100, *M*_n_ ≈ 2800 g/mol, 10 wt % PEO) was purchased from Sigma Aldrich (Saint Louis, MO, USA). The average composition of Pluronic L92 and Pluronic L81 can be represented as (EO)_8_(PO)_47_(EO)_8_ and (EO)_3_(PO)_50_(EO)_3_, respectively [22,29]. Monoethanolamine (MEA, ≥99.0%) was purchased from Sigma Aldrich. Carbon dioxide (99.7%) was purchased from AGA AB (Stockholm, Sweden). All chemicals were used as received. Milli-Q water was used as solvent (18.2 MΩ cm).

### 2.2. Loading of MEA with CO_2_

Monoethanolamine (MEA) was loaded following the procedure by Yang et al. [30]. A 25 wt % solution of MEA in Milli-Q water was prepared in a 100 mL three-necked round bottom flask. CO_2_ gas was bubbled through the amine solution for 7 h while the solution was stirred.

MEA is the most industrially important solvent for CO_2_ capture due to its low cost and the fast reaction kinetics with CO_2_ [6]. The reaction between MEA (here denoted as RNH_2_) and CO_2_ occurs according to the zwitterion mechanism [31]:

(1) $${\mathrm{CO}}_{2}+{\;\mathrm{RNH}}_{2}⇄{\;\mathrm{RNH}}_{2}{}^{+}{\mathrm{COO}}^{-}$$

In the first step of the reaction (Equation (1)), a zwitterion is formed. This zwitterion is deprotonated by a base (B) present in the system, which results in the formation of a carbamate (Equation (2)):

(2) $${\mathrm{RNH}}_{2}{}^{+}{\mathrm{COO}}^{-}+B⇄{\;\mathrm{RNHCOO}}^{-}+{\mathrm{BH}}^{+}$$

The base (B) that deprotonates the zwitterion can be water, OH^−^ ions, or MEA. When the base is MEA, the deprotonation of the carbamate can be written according to:

(3) $${\mathrm{RNH}}_{2}{}^{+}{\mathrm{COO}}^{-}+{\mathrm{RNH}}_{2}⇄{\;\mathrm{RNHCOO}}^{-}+{\mathrm{RNH}}_{3}{}^{+}$$

In that case, the overall reaction between MEA and CO_2_ can be written as:

(4) $${\mathrm{CO}}_{2}+2{\;\mathrm{RNH}}_{2}⇄{\;\mathrm{RNHCOO}}^{-}+{\mathrm{RNH}}_{3}{}^{+}$$

### 2.3. Total Inorganic Carbon (TIC) Analysis

Approximately 1 g of 25 wt % MEA solution was dissolved in 100 mL of Milli-Q water. TIC analysis was performed using a TOC-L Analyzer (Shimadzu, Kyoto, Japan) [32].

### 2.4. Sample Preparation

The samples were prepared by weighing the corresponding amounts of polymer, Milli-Q water, and monoethanolamine (MEA) into glass tubes. In the case of the samples containing CO_2_, previously CO_2_-loaded MEA was used. The glass tubes were flame-sealed and centrifuged in both directions at 2000 rpm for 15 min several times. The samples were then left to equilibrate for several weeks at room temperature.

### 2.5. Inspection under Polarized Light

The samples were visually inspected to determine the number of phases. In addition, the samples were examined between crossed polarizers to differentiate the isotropic phases (non-birefringent) from the anisotropic ones (birefringent).

### 2.6. Small-Angle X-ray Scattering (SAXS)

SAXS measurements were performed on a Bruker Nanostar SAXS system equipped with a Våntec-2000 detector (Bruker AXS GmbH, Karlsruhe, Germany). Κα radiation (λ = 1.54 Å) was provided by a IμS Cu microsource (Incoatec, Geesthacht, Germany) operating at 50 kV and 60 mA. The samples were placed in a sandwich cell with Kapton windows, and the measurements were performed at controlled temperature. Water was used as standard to calibrate the raw scattering data to absolute intensity scale. The scattering data was radially averaged to obtain the 1-D scattering profile as a function of the scattering vector. The scattering of the empty cell was subtracted from the corresponding measured sample. All experiments were conducted at 25 °C, and selected samples were also measured at 15, 35, and 45 °C.

The structure of the lyotropic liquid crystalline phases was determined from the relative positions of the SAXS diffraction peaks [22,24]. For the lamellar phases, the positions of the peaks follow the relationship 1:2:3:4… In the case of hexagonal structures, the positions of the peaks follow the ratios 1:√3:2:√7:3… The lattice parameters of the lamellar (Equation (5)) and hexagonal (Equation (6)) structures can be calculated as follows:

(5) $$q_{1}=\frac{2\pi }{d}$$

(6) $$q_{1}=\frac{4\pi }{a\sqrt{3}}$$

where *d* is the lamellar periodicity, *a* is the distance between centers of adjacent cylinders, and $q_{1}$ is the position of the first diffraction peak.

#### Definition of Polar and Apolar Domains and Calculation of the Interfacial Area

The polar and apolar domains of Pluronic/water/MEA were defined following the description by Alexandridis et al. [25,33]. L92 and L81 consist of 20 wt % and 10 wt % EO, respectively. To define the polar and apolar domains, we assume that the system is segregated. Therefore, the apolar volume fraction (*f*) can be defined as the volume fraction of the hydrophobic blocks of the polymer ($X\phi_{p}$). Similarly, the polar volume fraction (1 − *f*) can be defined as the volume fractions of water ($\phi_{w}$), MEA ($\phi_{MEA}$), and the hydrophilic blocks of the polymer ($(1-X)\phi_{p}$).

(7) $$f=X\phi_{p}$$

(8) $$1-f=(1-X)\phi_{p}+\phi_{w}+\phi_{MEA}$$

The calculated PPO volume fraction in the polymer (X) is 0.777 for L92, and 0.874 for L92. The bulk densities of L92, L81, MEA, and water have been used for the calculations (1.03, 1.03, 1.012, and 1.0 g/mL, respectively). The molecular volume ($\nu_{p}$) was calculated for L92 (5885 Å^3^) and L81 (4514 Å^3^).

The average interfacial area per polymer molecule ($\alpha_{p}$) can be calculated from the SAXS data [25,33]. For lamellar phases, the interfacial area can be calculated from the following equation:

(9) $$d=\frac{2\nu_{p}}{\phi_{p}\alpha_{p}}$$

For hexagonal aggregates, in order to obtain the interfacial area, the cylinder cross section radius ($R_{cyl}$) has to be calculated. The volume fraction of the cylinders ($\phi_{cyl}$) for oil-in-water structures is assumed to be equal to the apolar fraction of the polymer, *f*.

(10) Rcyl=a(32πϕcyl)12

The interfacial area of hexagonal liquid crystals can be obtained from:

(11) $$R_{cyl}=\frac{2f\nu_{p}}{\phi_{p}\alpha_{p}}$$

## 3. Results

### 3.1. Phase Behavior

**Figure 2:**
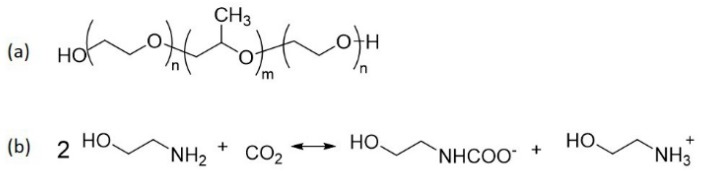
(**a**) Chemical structure of Pluronic. For L92 *n* = 8, *m* = 47. For L81, *n* = 3, *m* = 50; (**b**) Scheme of the overall reaction of MEA with CO_2_.

The phase behavior of the ternary system Pluronic L92/water/MEA was compared to the phase behavior of the same system using MEA loaded with CO_2_ (Pluronic L92/water/(MEA + CO_2_)). The CO_2_ loading of 25 wt % MEA determined by TIC was 0.51 mol CO_2_/mol MEA. Figure 2 shows the chemical structure of Pluronic and the overall reaction of MEA with CO_2_. For simplicity in the representation of the phase diagram, CO_2_-loaded MEA was considered as one component in the ternary system. The same study has been conducted using a more hydrophobic polymer (Pluronic L81) instead of Pluronic L92. However, it should be noted that Pluronic/water/(MEA + CO_2_) are not real ternary phase diagrams due to the presence of additional components formed when MEA reacts with CO_2_, namely carbamate and ammonium ions. In addition, it was not possible to distinguish between hexagonal and reverse hexagonal phases. Therefore, in this work the notation used for hexagonal phases (H) represents both hexagonal and reverse hexagonal phases.

#### 3.1.1. Phase Behavior of Pluronic L92/Water/MEA

**Figure 3:**
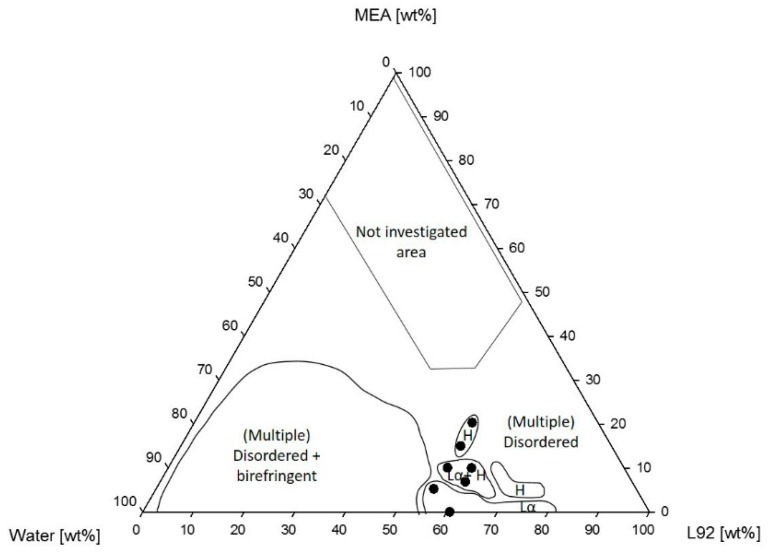
Phase diagram of Pluronic L92/water/MEA.

**Figure 4:**
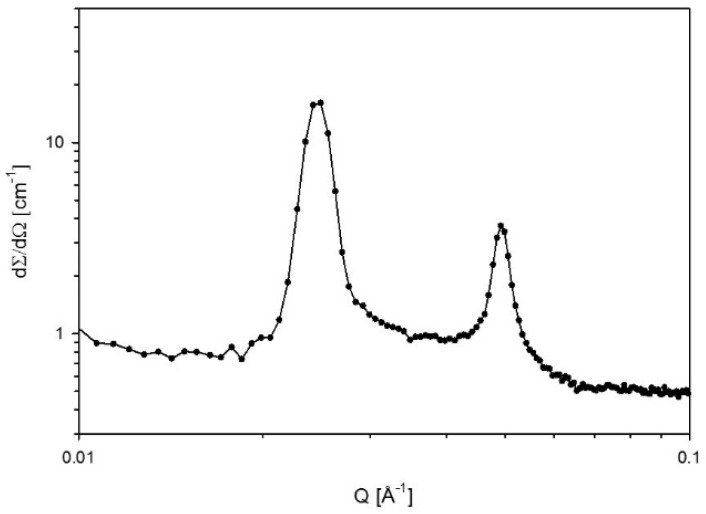
SAXS diffraction pattern obtained from a lamellar phase of a sample with composition 55% L92/5% MEA. The peak positions follow the ratio 1:2.

**Figure 5:**
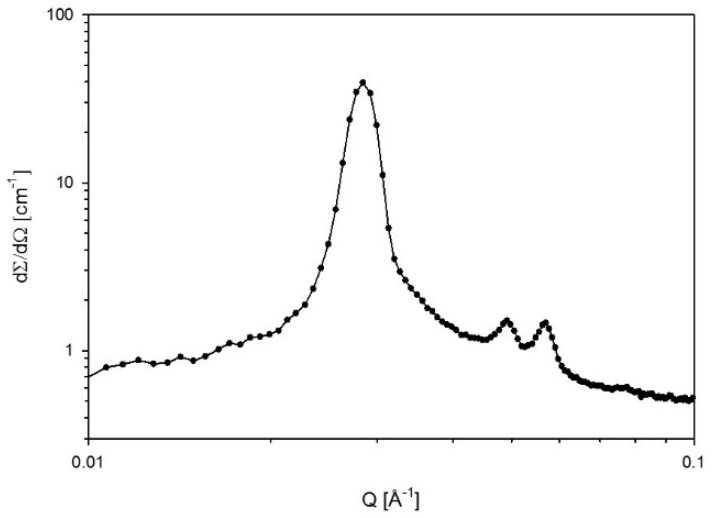
SAXS diffraction pattern obtained from a hexagonal phase of a sample with composition 75% L92/5% MEA. The peak positions follow the ratio 1:√3:2.

Figure 3 shows the phase diagram of the ternary system Pluronic L92/water/MEA obtained at 25 °C. The investigated areas of the phase diagram show a large region consisting of a mixture of disordered phases and a birefringent phase. Lamellar phases (Lα) were found at high polymer concentrations when only polymer/water were present in the sample, as previously reported by Svensson et al. [22]. The lamellar region was preserved when small amounts of MEA were present in the sample, although as the polymer composition increases, the addition of MEA led to the formation of coexisting lamellar and hexagonal phases, and at even higher polymer concentration, to a shift from lamellar to hexagonal phase. Further addition of MEA produces a shift from the lamellar + hexagonal region to hexagonal. No liquid crystalline phases were found above 20 wt % MEA. Above these concentrations, the samples consisted of several non-birefringent phases. Examples of a lamellar phase and a hexagonal phase are shown in Figure 4 and Figure 5, respectively.

**Figure 6:**
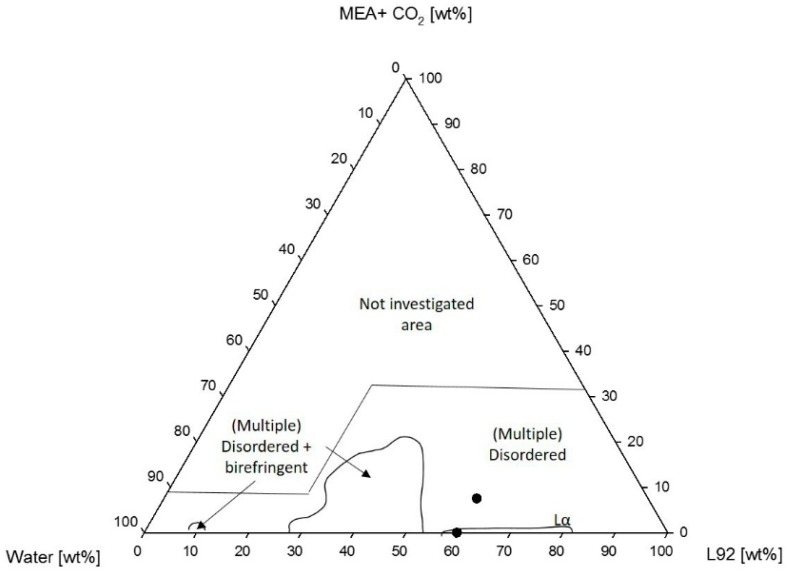
Phase diagram of Pluronic L92/water/MEA + CO_2_.

Similarly, the phase diagram of Pluronic L92/water/(MEA + CO_2_) is shown in Figure 6. Visual inspection of the samples showed that samples containing loaded MEA underwent a phase separation. At concentrations between 30 and 50 wt % L92, there was a region consisting of disordered and birefringent phases. The remaining investigated samples only consisted of phases without clear long range order, some of them with multiple phases.

#### 3.1.2. Phase Behavior of Pluronic L81/Water/MEA

**Figure 7:**
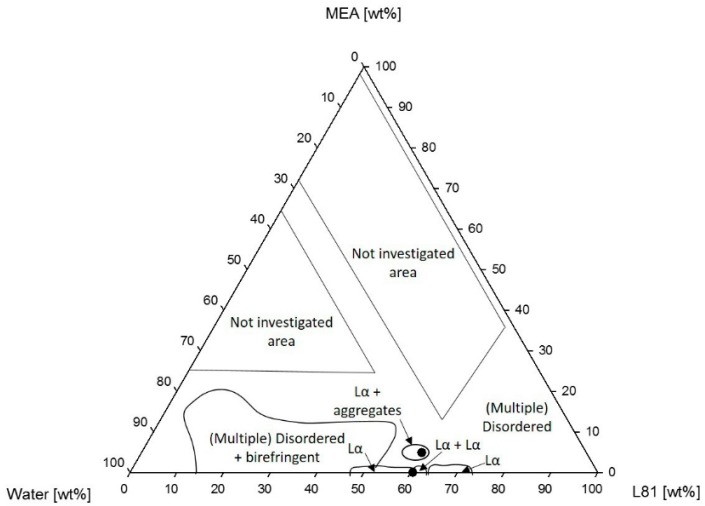
Phase diagram of Pluronic L81/water/MEA.

**Figure 8:**
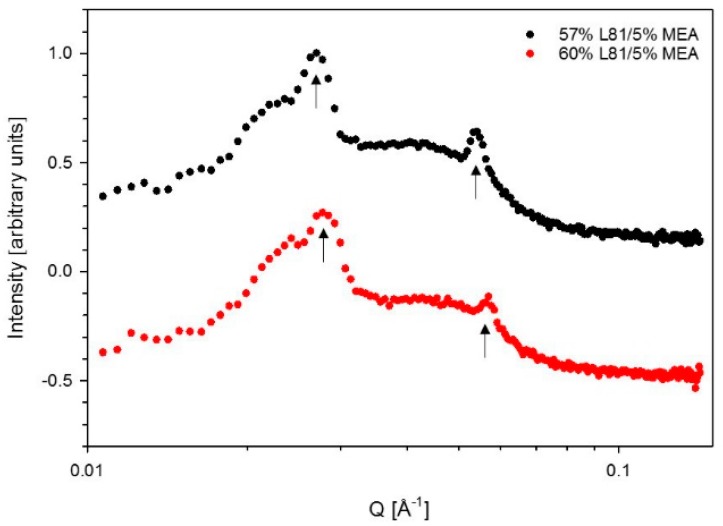
SAXS spectra of 57% L81/5% MEA and 60% L81/5% MEA. The arrows indicate the peaks following lamellar ratios of 1:2.

Figure 7 shows the phase diagram of Pluronic L81/water/MEA. The phase behavior of Pluronic L81/water has not been reported before. L81/water mixtures consisting of 50–70 wt % L81 were lamellar. However, at 62 wt %, a two-phase system was formed, consisting of two different lamellar phases, confirmed by the different lattice parameters of each phase shown in Table 1. In the investigated area, there was a large region consisting of a combination of disordered phases and birefringent phases. The addition of MEA to samples consisting of 55–70 wt % L81 led to phase separation and formation of isotropic phases. However, a small one-phase region consisting of a mixture of lamellar and a phase with no clear long range order was formed when the concentration of MEA was 5 wt %. The SAXS spectra of two samples within this region are shown in Figure 8.

**Figure 9:**
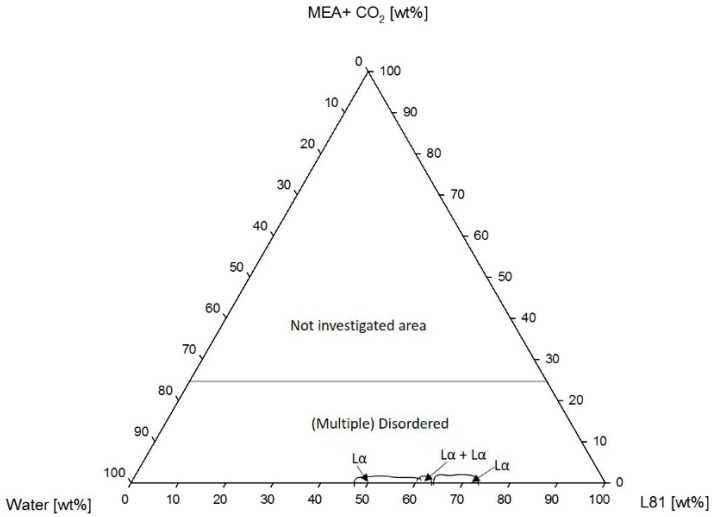
Phase diagram of Pluronic L81/water/MEA + CO_2_.

Figure 9 shows the corresponding phase diagram of L81 with CO_2_-loaded MEA. In this case, the presence of loaded MEA in the samples prevented self-assembly from occurring. The studied samples consisted of two or more disordered liquid phases.

### 3.2. Swelling

The swelling of the phases as MEA concentration increased was investigated to determine where MEA was found in the system. The samples were prepared at a constant copolymer composition to investigate the effect of replacing water by MEA in the system. Moreover, the interfacial area (a_p_) of the polymer molecules was calculated as well. The results are shown in Table 2.

In Figure 3, lines have been drawn to indicate the studied composition for the swelling experiments (55% and 60% L92). The lattice parameters of chosen samples along these lines have been calculated and are shown in Table 2. Temperature dependent measurements have been performed for samples in the 60% L92 line for samples with MEA. In the other cases, measurements were only performed at 25 °C. The samples were analyzed along the 55% line to study the effects of MEA in the swelling of the liquid crystalline aggregates. At 5% MEA a lamellar phase was formed. For 10% MEA, coexisting lamellar and hexagonal phases were formed with lattice parameters of 407 and 294 Å. As the MEA concentration increased to 15% and 20%, there was a phase transition to a hexagonal phase. The results showed that as MEA was added to the system, the swelling of the samples decreased, and, therefore, the interfacial area increased. The same behavior was observed for the samples along the 60% L92 line. At 15 °C, the samples formed a lamellar phase. Upon heating, the phase behavior changed to coexisting lamellar + hexagonal at 25 °C, and to hexagonal at 35 °C. In all cases, despite the temperature changes, the lattice parameters decreased with increasing MEA concentration. In the same way, the interfacial areas increased. Measurements were also performed at 45 °C; however, the results are not shown in Table 2. In the case of 7% MEA, the Bragg peaks in the spectrum recorded at 45 °C show the presence of an hexagonal phase together with an unidentified peak. This data point is not shown in Table 2, due to the lack of information about the coexisting microstructure. In the case of 10% MEA, the result is not shown because the microstructure is lost. In addition, the peaks at 45 °C were very broad because molecules are starting to disorganize. When samples with the same MEA concentration were compared (55% L92/10% MEA and 60% L92/10% MEA at 25 °C), the swelling of the aggregates decreased abruptly with only 5% polymer concentration increase.

The same analysis has been done in the case of L92/water/(MEA + CO_2_) (Figure 6). In Table 2, the result for 60% L92/(7% MEA + CO_2_) is shown. When water was replaced by CO_2_-loaded MEA, a “gel” consisting of two different lamellar phases was formed, and this gel was surrounded by a liquid phase. The lamellar spacings were 422 and 465 Å, and the corresponding interfacial areas were 47 and 42 Å^2^.

In the case of L81 (Figure 7), the swelling of the aggregates could only be studied along the 60% L81 dilution line because most samples phase-separated upon addition of MEA (both loaded and unloaded). The two studied samples, 0% and 5% MEA were within the lamellar region. However, dilution with MEA from 0% to 5% led to an increase of the lamellar spacing, and therefore the interfacial area decreases.

## 4. Discussion

### 4.1. Phase Behavior

#### 4.1.1. Phase Behavior of Pluronic L92/Water/MEA

In Figure 3, the lamellar region was found between 60 and 80 wt % L92 in the polymer water systems [22]. The addition of MEA induced a phase transition to a hexagonal phase, or to coexisting hexagonal and lamellar phases. However, above 20 wt % MEA, the system became disordered and macroscopically, the phases separate. At lower polymer concentrations, the system presented multiple phases in equilibrium, where one of them was visually birefringent. A similar behavior was observed by Ivanova et al. in ternary systems of Pluronic 105/water/polar cosolvent, where liquid crystalline regions are found along the polymer/water axis [27]. In the samples, there was a partitioning of MEA between the polymer-rich and polymer-lean phases, where the majority of MEA appears to be in the polymer-lean phase. The reasons behind phase separation are the low solubility of MEA in the polymer, and the relative polarities of the three components in the system. The poor solubility of MEA in L92 could be observed in the phase diagram, where the samples along the L92/MEA line formed disordered systems, in most cases with multiple phases. In addition, MEA was more polar than L92. Water has the highest polarity and will preferably interact with MEA and make the media more polar, and as a consequence induce phase separation [34,35]. When the samples were prepared using MEA loaded with CO_2_ (Figure 6), the phase behavior of the system changed. In this case, multiphase regions were formed, one of them consisting of disordered phases and a birefringent phase in equilibrium. In other words, the addition of loaded MEA favored phase separation, and prevented the formation of single-phase liquid crystalline phases. The reasoning behind this fact might be the presence of carbamate and ammonium ions formed after the reaction of MEA with CO_2_ [31], increasing the polarity of MEA, and favoring even more the interaction between MEA and water over the interaction between the polymer and water [34,35]. The variation in ionic strength in the system affects the self-assembly of the molecules into ordered microstructures, leading to phase separation of the samples, or in some cases inducing “gelation”. Like in the case of the phase diagrams with unloaded MEA, most of the samples presented polymer-rich and polymer-lean phases. MEA seemed to be found predominantly in the polymer-lean phases, due to its low solubility in L92.

#### 4.1.2. Phase Behavior of Pluronic L81/Water/MEA

A similar phenomenon was observed in the systems containing L81. Nevertheless, these systems were even more sensitive to the addition of MEA than the systems with L92. Pluronic L81 is a more hydrophobic polymer than L92, therefore, the miscibility of this polymer with polar solvents, such as water and MEA, is expected to be even lower than in the case of L92. The ternary phase diagram of L81/water/MEA (Figure 7) shows that the liquid crystalline region was essentially limited to the polymer/water line. There was a small region of coexisting lamellar and an unidentified phase when the concentration of MEA was 5 wt %. Unidentified peaks in the SAXS spectra (Figure 8) seem to correspond to correlation peaks, which indicate the presence of large disordered aggregates. Above this concentration of MEA, no liquid crystalline phases were found. In the same way as in the L92/water/MEA phase diagram, there was also a large region consisting of multiphase systems where one of the phases was liquid crystalline. However, the phase behavior of these regions was not within the scope of this work. As previously discussed for L92 systems, in the disordered and multi-phase regions, MEA was partitioned between the several phases. Due to the high polarity of MEA and the hydrophobic nature of L81, most MEA was found in the polymer-lean phases [34,35]. The equivalent phase diagram containing CO_2_-loaded MEA (Figure 9) shows that the addition of loaded MEA prevented the formation of ordered structures. In this phase diagram, no liquid crystalline regions were found except for the polymer/water line of the diagram. The presence of ions in the MEA solution has a stronger impact on the self-assembly of the system due to the increased hydrophobicity of L81 with respect to L92, and the increased polarity of CO_2_-loaded MEA compared with MEA [34,35]. However, the addition of unloaded MEA already hindered the self-assembly of the polymer chains, due to the high polarity of this solvent.

### 4.2. Swelling

The swelling of liquid crystalline phases has been studied at constant polymer composition. Two dilution lines (55% and 60% L92) have been drawn in the phase diagram of L92/water/MEA (Figure 3), and the lattice parameters and interfacial areas for the aggregates present in each phase have been calculated (Table 2). At 55% L92, a phase transition at constant polymer concentration can be observed as MEA concentration was increased. The transition from lamellar to hexagonal phase could be explained in terms of packing by the swelling of the PEO blocks by the cosolvent MEA, which leads to a decrease in the packing parameter. In other words, the addition of solvent to the systems favors the formation of structures with higher curvature and increases the interfacial area of the PEO blocks [27,28,36]. Due to the phase transitions occurring while diluting the sample with MEA, only the swelling results of two samples (15% MEA and 20% MEA) were comparable. The data from both sets of samples showed that within the hexagonal phase, with increasing MEA concentration, the lattice parameter size decreased. The decrease in the lattice parameters was caused by the increase of the interfacial area per molecule, which indicates that the PEO block or the PEO and PPO blocks were swollen by MEA [27,28].

A similar behavior was observed along the 60% line. The lattice parameters and interfacial areas of 7% MEA and 10% MEA were calculated at several temperatures. In all cases, the lattice parameters decreased abruptly with increasing MEA concentration, due to an increase in the interfacial area of the polymer molecules. As already discussed above, MEA appeared to be incorporated in the PEO blocks, increasing the interfacial area of the PEO blocks, and effectively decreasing the swelling [27,28]. Moreover, the two studied samples underwent a phase transition caused by temperature. At 25 °C, there was a phase transition from lamellar to coexisting lamellar and hexagonal phases; in other words, a more curved structure was formed upon addition of MEA [27,28]. The presence of coexisting lamellar and hexagonal phases indicates that MEA and water were partitioned between the two phases, one of them containing more MEA than the other. Moreover, in general for polar solvents, increased solvent content favors the formation of structures with positive curvature [27,28]. When samples with constant MEA concentration were compared (55% L92/10% MEA and 60% L92/10% MEA), the lattice parameters decreased at higher polymer concentration because the interface between polymer molecules and solvent increased, reducing the space between the polymer bilayers [33].

Regarding the system L92/water/(MEA + CO_2_) shown in Figure 6, the addition of CO_2_-loaded MEA favored phase separation. This is probably caused by the charged species present in CO_2_-loaded MEA, which increased the polarity difference between the apolar polymer and the polar solvents [34,35]. If 60% L92/0% MEA and 60% L92/(7% MEA + CO_2_) were compared, it can be seen in Table 2 that the spacings of the coexisting lamellar phases formed upon addition of CO_2_-loaded MEA were larger than the spacing of the sample without MEA. This result is in agreement with the previous explanation, which suggests that the increase of polarity of MEA when it is loaded increases its affinity for water [34,35]. Moreover, the sample consisted of a gel-like phase surrounded by a liquid phase. It should be noted that the phase behavior of both samples was different; therefore, it is not accurate to compare the lattice parameters of these two samples. The 60% L92/(7% MEA + CO_2_) can be compared to the equivalent sample of 60% L92/7% MEA at 25 °C. The phase behavior of both samples was different. The sample without CO_2_ formed lamellar + hexagonal phases, whereas the loaded sample formed two coexisting lamellar phases. The lamellar spacings of the sample containing CO_2_ were larger than the spacing of the unloaded sample, which might be a consequence of the higher polarity of loaded MEA compared to pure MEA, which reduced the affinity of water and MEA for the polymer. Nevertheless, as in the previous case, it is not accurate to compare the swelling of these two samples due to their different phase behavior.

The results obtained for L81/water/MEA (Table 2) show the opposite behavior than the previous results. The lattice parameters decreased when the samples were diluted with MEA [33]. The swelling was only calculated for one sample, due to phase separation of the system (Figure 7). The difference in behavior between L92 and L81 was caused by the different hydrophilicity of the polymers. L81 is less hydrophilic than L92 because it has shorter PEO blocks. When MEA is added to the system, it cannot swell the PEO shell of the aggregates because the PEO blocks are only three monomers long. In addition, during dilution with MEA, the concentration of water in the sample decreases, contributing to the shrinking of the PEO blocks, and decrease of the interfacial area [27,28]. It should be pointed out that the sample containing 5% MEA was described as a lamellar phase; however, from the SAXS spectrum shown in Figure 8, it can be seen that there are disorganized aggregates present in the sample as well. When CO_2_-loaded MEA was used as cosolvent (Figure 9), the difference in polarity between the two solvents and the polymer was so large that no liquid crystalline structures were formed.

The swelling experiments showed that in the case of L92, dilution of the samples with MEA led to an decrease in the lattice parameters of the lamellar and hexagonal phases [33]. This indicates that MEA was incorporated in the PEO blocks of the polymer, increasing the curvature of the aggregates with increasing MEA concentration [27,28]. This hypothesis is also in agreement with the observation that above 20% MEA, no liquid crystalline phases are found due to phase separation [34,35]. In the case of L81, the opposite behavior was observed due to the increased hydrophobicity of this polymer. The difference in polarity between the polymer and the solvents was so large, that the PEO blocks shrink, and the lattice parameters of the liquid crystalline phases [34,35]. Finally, the use of CO_2_-loaded MEA favored phase separation due to the charged species present in MEA. Consequently, the swelling of the aggregates increased [34,35].

## 5. Conclusions

In this work, the phase behavior of the ternary systems PEO-PPO-PEO/water/MEA has been investigated using unloaded and CO_2_-loaded MEA. Two different polymers have been studied: L92 and L81, and the structure of the liquid crystalline phases was determined by SAXS. It was observed that the use of CO_2_-loaded MEA (0.51 mol CO_2_/mol MEA) induced phase-separation of the samples. L92 was more promising for CO_2_ capture within the liquid crystals due to the higher solubility of MEA in this polymer than in L81. The swelling of the liquid crystalline phases was also investigated. For the L92/MEA system, the swelling of the samples decreased when the MEA concentration increased, suggesting that the PEO blocks were solvated by MEA. In the system L81/MEA, the swelling of the liquid crystalline samples increased with increasing MEA concentration due to the poor interactions between the cosolvent and MEA. Phase separation was favored in systems containing CO_2_-loaded MEA due to the increased polarity of MEA caused by the presence of carbamate and ammonium ions. In general, the results suggest that longer PEO blocks favored the formation liquid crystalline structures with MEA.

## Figures and Tables

**Table 1:** Lattice parameters of L81/water mixtures.

Composition	Phases	Lattice Parameters (Å)
50% L81	1 phase: Lα	196
55% L81	1 phase: Lα	196
60% L81	1 phase: Lα	188
62% L81	2 phases: Lα, Lα	188, 205
65% L81	1 phase: Lα	184
70% L81	1 phase: Lα	173

**Table 2:** Phases, lattice parameters, and interfacial areas of the birefringent samples along the dilution lines.

Polymer (*w*/*w*)	MEA (*w*/*w*)	15 °C			25 °C			35 °C		
		Phases	*a*, *d* (Å)	*a_p_* (Å^2^)	Phases	*a*, *d* (Å)	*a_p_* (Å^2^)	Phases	*a*, *d* (Å)	*a_p_* (Å^2^)
55% L92	5%				Lα	252	86			
	10%				Lα + H	*a* = 407; *d* = 294	H = 81; Lα = 73			
	15%				H	340	68			
	20%				H	269	100			
60% L92	0%				Lα	221	89			
	7%	Lα	221	89	Lα + H	*a* = 239; *d* = 300	H = 85; Lα = 82	H	340	75
	10%	Lα	121	164	Lα + H	*a* = 126; *d* = 173	H = 148; Lα = 157	H	176	146
60% L92	7% + CO_2_				Lα + Lα	422; 465	47; 42			
60% L81	0%				Lα	188	80			
	5%				Lα	221	69			
